# A New Mutation Affecting FRQ-Less Rhythms in the Circadian System of *Neurospora crassa*

**DOI:** 10.1371/journal.pgen.1002151

**Published:** 2011-06-23

**Authors:** Sanshu Li, Kamyar Motavaze, Elizabeth Kafes, Sujiththa Suntharalingam, Patricia Lakin-Thomas

**Affiliations:** 1 Department of Biology, York University, Toronto, Canada; 2 Department of Microbiology, Tehran North Branch, Islamic Azad University, Tehran, Iran; Texas A&M University, United States of America

## Abstract

We are using the fungus *Neurospora crassa* as a model organism to study the circadian system of eukaryotes. Although the FRQ/WCC feedback loop is said to be central to the circadian system in *Neurospora*, rhythms can still be seen under many conditions in FRQ-less (*frq* knockout) strains. To try to identify components of the FRQ-less oscillator (FLO), we carried out a mutagenesis screen in a FRQ-less strain and selected colonies with altered conidiation (spore-formation) rhythms. A mutation we named UV90 affects rhythmicity in both FRQ-less and FRQ-sufficient strains. The UV90 mutation affects FRQ-less rhythms in two conditions: the free-running long-period rhythm in choline-depleted *chol-1* strains becomes arrhythmic, and the heat-entrained rhythm in the *frq^10^* knockout is severely altered. In a FRQ-sufficient background, the UV90 mutation causes damping of the free-running conidiation rhythm, reduction of the amplitude of the FRQ protein rhythm, and increased phase-resetting responses to both light and heat pulses, consistent with a decreased amplitude of the circadian oscillator. The UV90 mutation also has small but significant effects on the period of the conidiation rhythm and on growth rate. The wild-type UV90 gene product appears to be required for a functional FLO and for sustained, high-amplitude rhythms in FRQ-sufficient conditions. The UV90 gene product may therefore be a good candidate for a component of the FRQ-less oscillator. These results support a model of the *Neurospora* circadian system in which the FRQ/WCC feedback loop mutually interacts with a single FLO in an integrated circadian system.

## Author Summary

All eukaryotes (including humans), and some bacteria, have evolved internal biological clocks that control activity and physiology in a daily (circadian) cycle. The molecular oscillators that drive these circadian rhythms are said to depend on rhythmic expression and feedback regulation of a small set of “clock genes.” However, there is increasing evidence that there is more to the story than these well-studied feedback loops. In the fungus *Neurospora crassa*, rhythms can still be seen in mutants that are missing one of the clock genes, *frq*. There is currently a controversy as to whether there are many different *frq-*less oscillators and whether they interact with the *frq* clock. To identify the molecular mechanism that drives these *frq*-less rhythms, we started with a *frq-*less strain and mutagenized it to look for genes that affect the *frq-*less rhythms. We found a new mutation that not only disrupted two *frq*-less rhythms but also affected the rhythm when the *frq* gene is present. Our results suggest there is only one *frq-*less oscillator, and it interacts with the *frq* clock. Our new mutation may identify a gene that is critical to both oscillators. We suggest that a similar clock architecture may be common to all organisms.

## Introduction

Circadian rhythms are approximately 24 h cycles of behavior, physiology, etc. that are driven by an endogenous biological clock. They are found at all levels of eukaryotic life, and in some prokaryotes. Molecular models for the endogenous oscillators that drive these rhythms in eukaryotes are based on rhythmic transcription of a small number of “clock genes”, translation of the rhythmic transcripts into rhythmic levels of clock proteins, and negative regulation of the clock genes by their own proteins. These models have been called transcription/translation feedback loops (TTFL).

Although a great deal of evidence has accumulated to support the concept of negative and positive feedback loops regulating the expression of these clock genes, there are many indications that the TTFL model is inadequate as a complete clock mechanism (reviewed in [1]). In recent mammalian examples, it was found that cycling of mammalian clock proteins CLOCK and CRY is not required for rhythmicity in fibroblasts [2]; large reductions in overall transcription rate and levels of clock proteins do not eliminate circadian oscillations in mouse fibroblasts [3]; and rhythmic cAMP signaling is required to sustain rhythmic transcription in mammalian cells [4]. These and other findings are not compatible with the canonical TTFL models.

In the prokaryotic cyanobacterium *Synechococcus elongatus*, a post-translational oscillator has been shown to be the core pacemaker. It can operate *in vitro* in the absence of any transcription or translation, but *in vivo* a TTFL is coupled to it as a slave oscillator [5], [6]. The architecture of the cyanobacterial clock provides certain advantages that may be relevant to eukaryotic clock systems as well [6]. Progress in understanding circadian oscillators in eukaryotes will depend on identifying clock components outside of the TTFL [7], [8].

The filamentous fungus *Neurospora crassa* is a model organism that has provided many insights into the molecular basis of circadian rhythmicity [9]–[11]. Asexual spore formation (conidiation) is controlled by the circadian clock in *Neurospora*, and rhythmic spore formation can be easily monitored during growth as a pattern of thick conidiation “bands” alternating with thin growth in “interbands” as the fungal mycelium advances across a solid agar surface. The current model for the *Neurospora* circadian oscillator (called the FRQ/WCC TTFL) consists of interlocked negative and positive feedback loops involving the clock genes *frq*, *wc-1* and *wc-2* [9], in which a complex of the WC-1 and WC-2 proteins (WCC) activates transcription of *frq* and is in turn negatively regulated by FRQ protein.

However, there have been many reports of rhythmicity with periods in the circadian range that can be seen in strains with null mutations in *frq* or *wc* genes. These include conidiation rhythms [12]–[22] and molecular/biochemical rhythms [23]–[26]. These rhythms are said by some to be driven by many different “FRQ-less oscillators” (FLOs) that may be completely separate from the FRQ/WCC feedback loop [26]–[28]. An alternative view sees the FRQ/WCC TTFL as a component of a larger circadian system in which the FRQ/WCC TTFL interacts with a single FLO [29]. *Neurospora* provides a unique eukaryotic system in which we can directly access clock components outside of the TTFL by assaying rhythmicity in FRQ-less strains in which the TTFL is not functioning. We are using this system to identify components of FLO by searching for mutations affecting rhythmicity in FRQ-less strains. Such mutations can also provide insight into the interactions between the FRQ/WCC TTFL and the FLO. The new mutation we report here disrupts two FRQ-less rhythms and also affects the FRQ/WCC TTFL, suggesting that a single FLO may interact with the FRQ/WCC TTFL in a circadian architecture similar to that of cyanobacteria.

## Results

### Isolation and initial characterization of a mutation affecting FRQ-less rhythmicity

**Figure 1:**
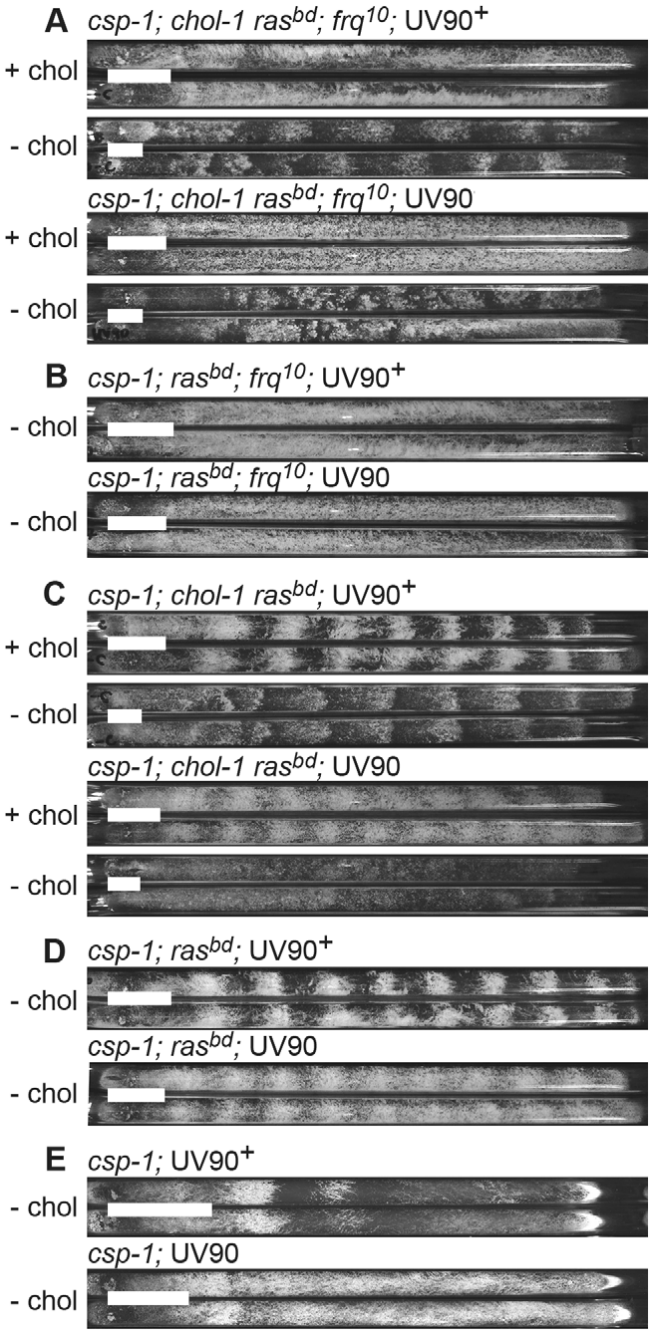
Phenotypes of UV90 mutants. Cultures were grown at 22°C in DD in race tubes on MA medium with (+ chol) or without (−chol) 100 µM choline. Growth was from left to right. White bars represent an average distance for 24 h of growth. Two sibling strains from a cross are shown in pairs. All strains carry the *csp-1* mutation. A–D: Progeny are from the backcross of *csp-1*; *chol-1 ras^bd^*; *frq^10^*; UV90 to *ras^bd^*. A. All strains carry the *ras-1^bd^*, *frq^10^* and *chol-1* mutations and were grown with or without choline. B. All strains carry the *ras-1^bd^* and *frq^10^* mutations and were grown without choline. C. All strains carry the *ras-1^bd^* and *chol-1* mutations and were grown with or without choline. D. All strains carry the *ras-1^bd^* mutation and were grown without choline. E. Progeny are from the cross of *csp-1*; *ras^bd^*; UV90 to Mauriceville. All strains were grown without choline.

To isolate mutations affecting the FLO, we conducted a mutagenesis screen to identify mutations affecting conidiation rhythms in a *frq* knockout background. We used UV light to mutagenize uninucleate microconidial spores of a strain carrying both the *frq^10^* knockout allele and *chol-1*. Strains carrying the *chol-1* mutation express conidiation rhythms in FRQ-less strains on choline-deficient media, and these rhythms are therefore an example of a FLO [16]. On high choline, *chol-1* strains are identical to *chol^+^* [16]. The parent strain for mutagenesis was both *frq^10^* and *chol-1* and was therefore rhythmic without choline but arrhythmic on high choline (Figure 1A, upper 4 tubes). We analyzed 600 colonies derived from mutagenized spores on individual race tubes and found one mutant that was arrhythmic both with and without choline (Figure 1A, lower 4 tubes). The mutation was named UV90, as it was the 90^th^ spore analyzed after UV mutagenesis.

This strain, genotype *csp-1*; *chol-1 ras^bd^*; *frq^10^*; UV90, was backcrossed to *ras^bd^* and the phenotypes of the progeny were analyzed. The *chol-1* phenotype was assayed by comparing growth rates on choline-supplemented and choline-deficient media. The conidiation banding phenotypes were easily identified among the progeny and these phenotypes were consistently identified by two individuals using blind-coded tubes or photographs. Periods and growth rates of all strains described below are listed in Table 1, and conidiation phenotypes are presented in Figure 1.

**Table 1:** Periods and growth rates of UV90 wild-type and mutant strains.

		UV90^+^		UV90	
Genotype	Choline	Period (h)	Growth rate (mm/h)	Period (h)	Growth rate (mm/h)
*csp-1; chol-1 ras^bd^; frq^10^*	+	arrhythmic	1.28±.01 (8)	arrhythmic	1.19±.01 (12)
	−	54.3±2.27 (32)	0.71±.01 (32)	arrhythmic	0.71±.03 (12)
*csp-1; ras^bd^; frq^10^*	+	arrhythmic	1.28±.02 (11)	arrhythmic	1.16±.02 (13)
	−	arrhythmic	1.34±.02 (11)	arrhythmic	1.19±.02 (13)
*csp-1; chol-1 ras^bd^*	+	21.2±.08 (26)	1.18±.01 (26)	21.6±.20 (16)	1.07±.01 (16)
	−	39.9±1.40 (20)	0.69±.01 (20)	arrhythmic	0.66±.02 (10)
*csp-1; ras^bd^*	+	21.0±.08 (26)	1.22±.01 (26)	22.2±.40 (15)	1.10±.01 (15)
	−	21.2±.06 (20)	1.29±.02 (20)	21.1±.73 (9)	1.16±.02 (9)
*csp-1*	−	22.0±.44 (20)	2.12±.09 (21)	N.D.	1.65±.03 (21)

Cultures were grown in DD at 22°C, with (+) or without (−) 100 µM choline. Values are mean ± S.E.M. (number of race tubes). Where a period is reported, all tubes were rhythmic, except for *csp-1* UV90^+^ in which one tube was unclear. N.D. = not determined; too few bands were visible to accurately calculate periods.

**Figure 2:**
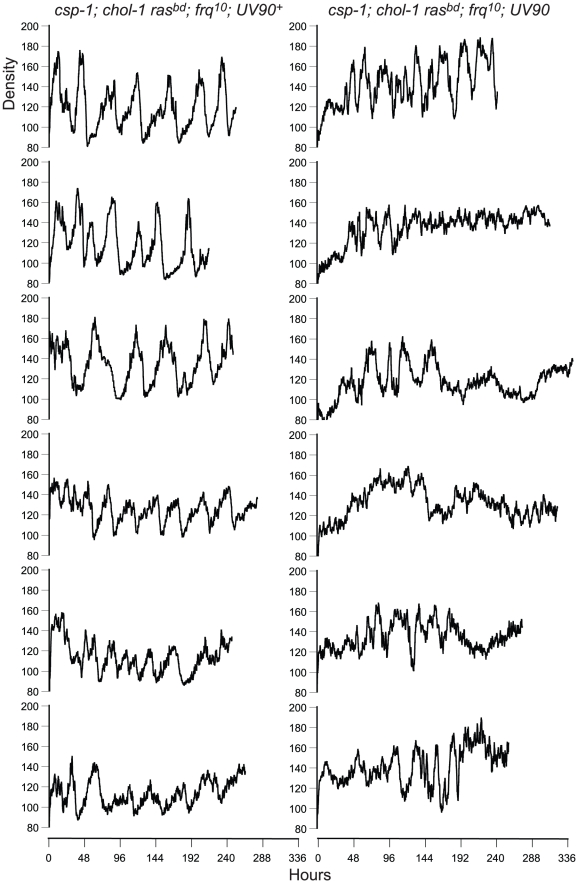
Densitometry of *chol-1*; *frq^10^* progeny. Race tube cultures of *csp-1*; *chol-1 ras^bd^*; *frq^10^* progeny from the backcross of *csp-1*; *chol-1 ras^bd^*; *frq^10^*; UV90 to *ras^bd^* were analyzed by densitometry. Six progeny from each class were randomly chosen for analysis. Left column: UV90^+^ progeny. Right column: UV90 progeny. All cultures were grown without choline. Density is in arbitrary units. Time is in hours after transfer from LL (constant light) to DD.

The *chol-1* progeny were identified by slow growth on low choline medium and rapid growth on high choline (Table 1). Among the *chol-1* progeny, the *frq^10^*; UV90 double mutant phenotype was identified by its similarity to the mutant parent: short aerial hyphae with very even conidiation on high choline (Figure 1A, tubes 5 and 6) and heavier conidiation and disrupted rhythmicity on low choline (Figure 1A, tubes 7 and 8). Densitometry traces of individual *chol-1*; *frq^10^* progeny are presented in Figure 2. The *chol^+^* progeny were identified by rapid growth on both high and low choline (Table 1). Among the *chol^+^* progeny, the *frq^10^*; UV90 double mutants (Figure 1B) were identified as having a phenotype on choline-free medium that was identical to the *chol-1*; *frq^10^*; UV90 parent strain on high choline (Figure 1A, tubes 5 and 6).

**Figure 3:**
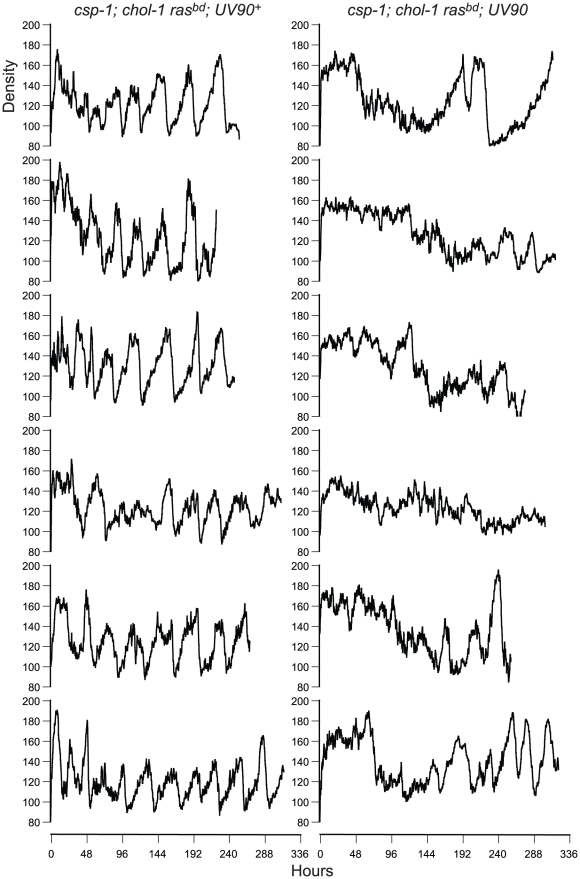
Densitometry of *chol-1*; *frq^+^* progeny. Race tube cultures of *csp-1*; *chol-1 ras^bd^* progeny from the backcross of *csp-1*; *chol-1 ras^bd^*; *frq^10^*; UV90 to *ras^bd^* were analyzed by densitometry. All other conditions as in Figure 2.

When the *frq^10^* mutation was crossed out of the original UV90 strain, the effects of the UV90 mutation on the circadian system when the FRQ/WCC oscillator is functional could be observed. The *chol-1*; *frq^+^* progeny (Figure 1C) were identified by the presence of rhythmicity on high choline (Figure 1C, tubes 1, 2, 5 and 6). Progeny in which the long-period rhythm on low choline was abolished (Figure 1C, tubes 7 and 8) were identified as UV90. Densitometry traces of individual *chol-1*; *frq^+^* progeny are presented in Figure 3. These progeny also showed a low-amplitude rhythm on high choline (Figure 1C, tubes 5 and 6).

**Figure 4:**
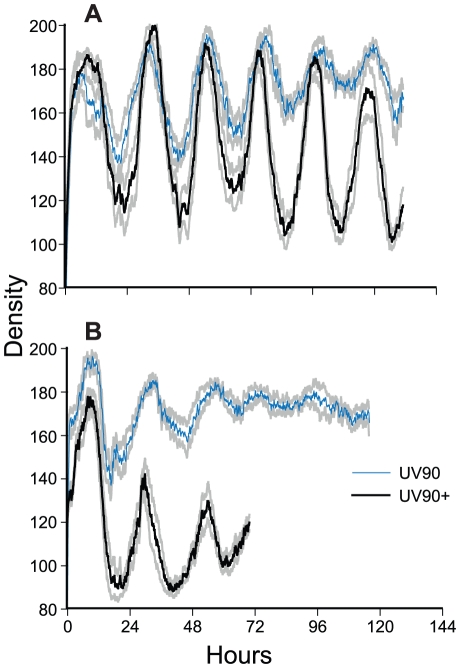
Densitometry of *chol^+^*; *frq^+^* progeny. Race tube cultures of progeny from the cross of *csp-1*; *ras^bd^*; UV90 to Mauriceville were analyzed by densitometry. Six *csp-1* progeny from each class were randomly chosen for analysis. Densitometry traces were averaged for each class and plotted ± one S.E.M. A. *ras^bd^* progeny. B. *ras^+^* progeny. Thick black lines: UV90^+^ progeny. Thin blue lines: UV90 progeny. Gray lines: ± one S.E.M. All cultures were grown without choline. Density is in arbitrary units. Time is in hours after transfer from LL to DD.

The *chol^+^*; *frq^+^* progeny (Figure 1D) were identified as indistinfguishable on choline-deficient medium from the phenotypes of the *chol-1* progeny on high choline (Figure 1C, tubes 1, 2, 5 and 6). The UV90 progeny were identified by the damped conidiation rhythm (Figure 1D, tubes 3 and 4). The low amplitude rhythm of *frq^+^* UV90 strains was due to an increase in average levels of conidiation, as shown by densitometry (Figure 4A).

One of the *csp-1*; *ras^bd^*; UV90 progeny was backcrossed to the Mauriceville wild-type to remove the *ras^bd^* mutation. The *ras^+^* progeny were identified by growth rates more rapid than the *ras^bd^* strains (Table 1). Although these progeny (Figure 1E) produced very poor banding rhythms, as expected, a UV90 phenotype was noticeable, increasing conidiation levels and producing nearly constant conidiation (Figure 1E, tubes 3 and 4, and Figure 4B).

We conclude that the UV90 mutation severely compromises the function of the FLO in *chol-1*, completely disrupting conidiation rhythmicity in the *frq^10^* knockout background in choline-deficient conditions. UV90 also damps the amplitude of the conidiation rhythm in *frq^+^* strains in the presence of a functional FRQ/WCC TTFL.

The UV90 phenotype was found to segregate in backcrosses with the expected 1∶1 ratio for segregation of a single-gene mutation. In the backcross of the original *csp-1*; *chol-1 ras^bd^*; *frq^10^*; UV90 mutant to *ras^bd^*, out of 88 *csp-1*; *ras^bd^* progeny assayed, 44 were classified as UV90 and 44 as UV90^+^ based on banding phenotypes on high and low choline. In the cross to Mauriceville of a putative *csp-1*; *ras^bd^*; UV90 strain, out of 40 *csp-1*; *ras^bd^* progeny assayed, 21 were classified as UV90 and 19 were classified as UV90^+^ based on banding phenotype.

**Figure 5:**
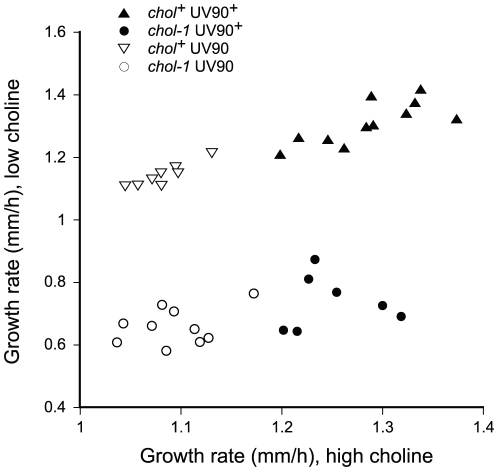
Segregation of growth rates. Progeny from the backcross of *csp-1*; *chol-1 ras^bd^*; *frq^10^*; UV90 to *ras^bd^* were grown at 22°C in DD in race tubes on MA medium with 100 µM choline (high choline) or without added choline (low choline) and growth rates were determined. All progeny carry the *csp-1*, *frq^+^* and *ras^bd^* alleles. Note the change in scales between the X and Y axes.

Among the *chol-1* progeny of the backcross of the original mutant to *ras^bd^*, the two UV90 banding phenotypes segregated together. Out of 45 *csp-1*; *chol-1 ras^bd^* progeny tested, 39 gave clear phenotypes on choline-deficient medium and 6 were unclear. Of those 39 clear phenotypes, 20 produced bands on low choline similar to UV90^+^ and all had high-amplitude banding rhythms on high choline; 19 were arrhythmic on low choline and all produced low-amplitude bands on high choline similar to UV90. The UV90 mutation slightly decreased the growth rates in most cases (Table 1). This property also co-segregated with the UV90 banding phenotypes. Figure 5 presents growth rate data for *frq^+^* progeny grown on high and low choline media, and it can be seen that progeny with a UV90^+^ banding phenotype, both *chol^+^* and *chol-1*, clustered together at higher growth rates than UV90 progeny.

The UV90 mutation mapped to the right arm of linkage group (LG) VI. In the backcross of the original UV90 strain to *ras^bd^*, the UV90 phenotype was unlinked to either *frq* (LG VIIR) or *chol-1* (LG IVR): out of 88 progeny tested, 50.0% were recombinant with *frq* and 48.9% were recombinant with *chol-1*. In a cross to the multiply-marked *alcoy* tester strain [30], recombination frequencies were: 51.9% with *cot-1* (LG IVL/VR), 40.7% with *al-1* (LG IR/IIL) and 16.7% with *ylo-1* (LG IIIR/VIR). These results suggested that UV90 is linked to either LG III or LG VI. The original UV90 strain was crossed to the Mauriceville wild-type and mapped using cleaved amplified polymorphic sequence (CAPS) markers and bulked segregant analysis according to the method of Jin *et al.* [31]. The UV90 phenotype was unlinked to a CAPS marker on LG III (3–52-EcoRI in Jin *et al*.) and was linked to CAPS markers 6–39-HaeII on the left of the centromere on LG VI, and 6–68-MspI on the right of the centromere. Recombination rates between UV90 and 6–39-HaeII were roughly 5–10% (estimated from the relative band intensities of the cleaved PCR fragments from the bulked segregants) and recombination between UV90 and 6–68-MspI was roughly 0–5%, suggesting that UV90 maps to the right arm of LG VI, in a gene-rich region of the chromosome.

### Effects of UV90 on growth rate and temperature compensation of period

**Figure 6:**
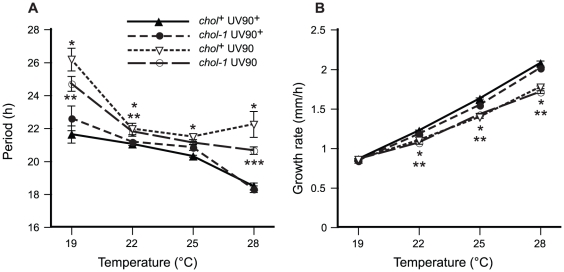
Effects of UV90 mutation on growth rate and temperature compensation of period. The period of the conidiation rhythm and the growth rate were assayed at different constant growth temperatures on medium containing 100 µM choline. Means are plotted ± one S.E.M., N = 6–26. Closed symbols: UV90^+^ strains. Open symbols: UV90 strains. Triangles: *chol-1^+^* strains. Circles: *chol-1* strains. All strains carry the *ras^bd^* and *csp-1* mutations and are wild-type for *frq*. A. Period of the conidiation rhythm, in hours. *: *chol^+^* UV90 significantly different from *chol^+^* UV90^+^, p<0.01. **: *chol-1* UV90 significantly different from *chol-1* UV90^+^, p<0.05. ***: *chol-1* UV90 significantly different from *chol-1* UV90^+^, p<0.01. B. Growth rate, in mm/h. Note that most S.E.M.s are smaller than the plot symbols. *: *chol^+^* UV90 significantly different from *chol^+^* UV90^+^, p<0.01. **: *chol-1* UV90 significantly different from *chol-1* UV90^+^, p<0.01.

One of the defining characteristics of a circadian rhythm is that the period of the rhythm does not change appreciably when the organism is maintained at different constant temperatures; this property of “temperature compensation” distinguishes circadian clocks from simple chemical reactions that increase in rate with an increase in temperature. We assayed the conidiation rhythm of our new mutant strain at different constant growth temperatures to determine the effects of the UV90 mutation on period and temperature compensation of the rhythm. All strains were wild-type for *frq*. Both *chol^+^* and *chol-1* strains were assayed on high choline to repair the defect in *chol-1*. As shown in Figure 6A, the UV90 mutation significantly increased the period by several hours at some temperatures, and altered the response of the period to temperature in comparison to the UV90^+^ strains. The UV90 mutation also decreased the growth rate by small but significant amounts at temperatures above 19°C (Figure 6B). These results suggest that the UV90 mutation may have minor effects on metabolism affecting growth, and minor but significant effects on the temperature compensation of the circadian clock in the presence of a functional TTFL.

### Effects of UV90 on entrainment to heat pulses

**Figure 7:**
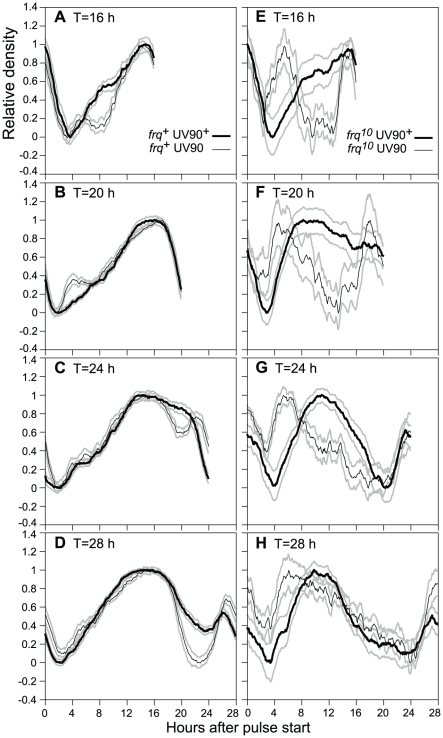
Effects of UV90 mutation on entrainment to high temperature pulses. All strains carry the *ras-1^bd^*, *csp-1* and *chol-1* mutations and were grown on high choline to repair the *chol-1* defect. Cultures were grown in DD at 22°C and were subjected to 2-h 32°C pulses at repeated intervals of T hours (16, 20, 24 or 28 h), in which T is the length of one cycle from one pulse start to the next pulse start. Culture density was determined and the last two complete cycles on each race tube were averaged for 6 race tubes. Density traces were normalized and are plotted ± one S.E.M., N = 12, against hours after the beginning of the 2-h heat pulse. Thick black lines: wild-type for UV90; thin black lines: UV90 mutant; grey lines: ± S.E.M. A–D. Comparison of UV90 wild-type with UV90 mutant in the *frq^+^* background. E–H. Comparison of UV90 wild-type with UV90 mutant in the *frq^10^* background.

It has been shown that FRQ-less strains can be entrained to repeated pulses of high temperature and behave as if they contain a functional heat-entrainable oscillator [15], . This heat-entrainable oscillator is a second example of a FLO. We used 2-hour pulses of 32°C on cultures growing at 22°C to entrain the conidiation rhythm to various T-cycles (where T = total number of hours in the cycle) (Figure 7). Changes in peak timing and peak shape in different T-cycles are indicative of entrainment of an underlying oscillator [19], [32]. In the presence of wild-type *frq* (Figure 7A–7D), the UV90 mutation had small but significant effects on the shape of the entrained peaks. In the FRQ-less background (Figure 7E–7H), the UV90 mutation had a dramatic effect on the peak timing, shifting the major peak to a much earlier time. We conclude that the UV90 mutation has a greater effect on entrainment behavior in the absence of functional FRQ (in the *frq^10^* strain) than in the presence of functional FRQ, and therefore is likely to primarily affect the FLO rather than the FRQ/WCC feedback loop.

### Effects of UV90 on phase-resetting

The damping of the amplitude of the conidiation rhythm seen in UV90 mutant strains with functional FRQ (Figure 1D) could be due to either an effect on the output from the circadian oscillator, such as continuous activation of the conidiation developmental pathway, or an effect on the amplitude of the circadian oscillator itself. To distinguish between these two possibilities, the amplitude of the oscillator was probed with phase-resetting stimuli. According to oscillator theory [33], [34], an oscillator with a small amplitude will respond to a particular stimulus with a larger phase resetting response than will an oscillator with a large amplitude. A similar effect should be seen with any type of stimulus, regardless of the input pathway it uses [33].

**Figure 8:**
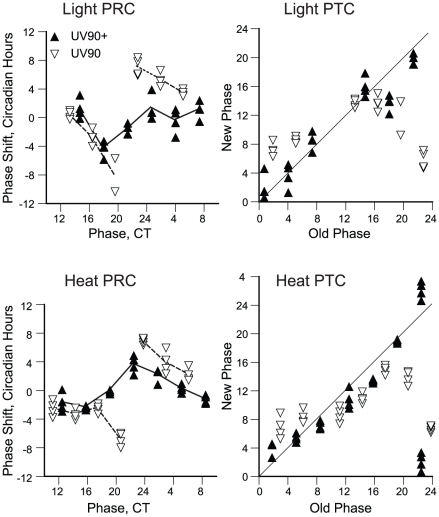
Effects of UV90 mutation on phase-resetting. All strains carry the *ras-1^bd^* and *csp-1* mutations. Cultures were grown in DD at 22°C. Solid triangles: UV90^+^. Open triangles: UV90. Each point represents an individual race tube. Top row: Resetting by 2 min light pulses. Bottom row: Resetting by 15 min heat pulses. Left column: Phase response curves, plotting the change in phase (phase shift in circadian hours) against the circadian time (CT) of the pulse. Average phase shifts are connected by lines. Right column: Phase transition curves, plotting the new phase after the pulse in circadian hours against the circadian time of the pulse (“old phase”). Diagonal lines indicate the locus of “no effect” where new phase equals old phase. Note that the UV90^+^ data for old phase 22.5 in the heat PTC have been double-plotted to show the continuity of the data.

We used both light pulses and high temperature pulses to probe the amplitude of the oscillator in the UV90 mutant strains in the *frq^+^ chol^+^* background. Data were plotted in both the traditional phase response curve (PRC) format, plotting phase shifts against the circadian time of the pulse, and in the phase transition curve (PTC) format [35], plotting new phase against old phase. The PTC format is preferred, as it does not introduce artifactual breaks in the data where large phase delays meet large phase advances [36]. As seen in Figure 8, the PRCs for the UV90 mutant showed larger phase shifts than the wild-type for both light and heat stimuli. The PTCs indicate that under these conditions, the wild-type produced type 1 PTCs and the UV90 mutant produced type 0 PTCs. A type 1 PTC is indicative of a weaker response than a type 0 PTC [34]–[36]. Because the same stimuli produced a weak response from wild-type and a stronger response from the UV90 mutant, these results are consistent with a smaller oscillator amplitude for the UV90 mutant [36].

### Effects of UV90 on expression of FRQ protein

**Figure 9:**
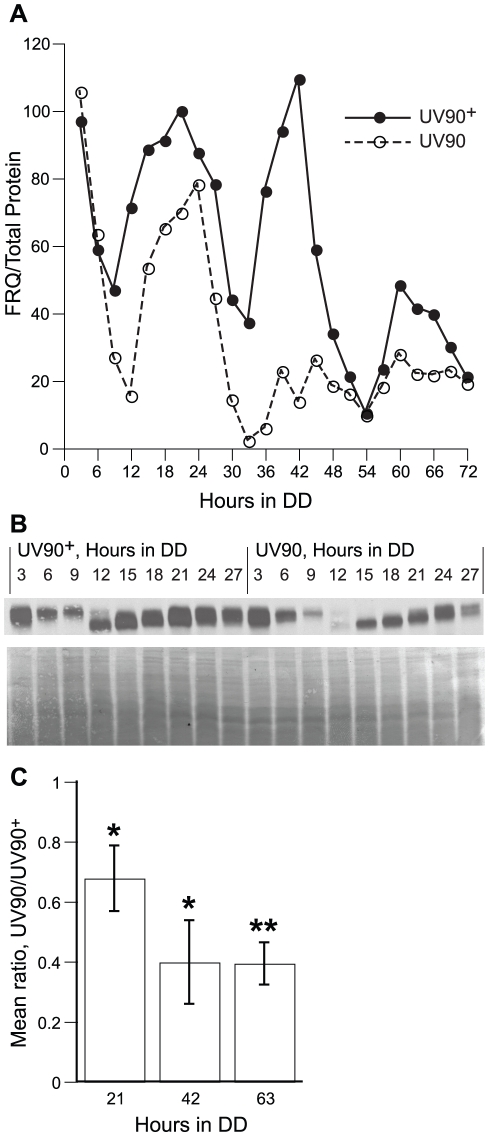
Effects of UV90 mutation on expression of FRQ protein. Both strains carry the *ras-1^bd^*, *csp-1* and *chol-1* mutations and were grown on high choline to repair the *chol-1* defect. Cultures were grown in DD for varying lengths of time and protein extracts were assayed for FRQ protein by immunoblotting. A. Relative level of FRQ, normalized to total protein by Coomassie staining. Solid circles: UV90 wild-type; open circles: UV90 mutant. Points are from a single series of experiments; similar results were obtained in a replicate experiment. B. Upper panel: Immunoblotting of FRQ protein for one cycle, 3–27 h in DD, corresponding to the time-series in A. UV90 wild-type samples are on the left half and UV90 mutant samples on the right half of the blot. Lower panel: Coomassie stained membrane of samples in upper panel. C. Ratios of relative FRQ levels in UV90 vs. UV90^+^. Ratios were calculated at three peak time points (21, 42 and 63 hours in DD) in three independent experiments and mean ratios are plotted ± one S.E.M. *: significantly less than 1.00, p<0.05. **: significantly less than 1.00, p<0.01.

We assayed the relative levels of FRQ protein in the UV90 mutant strain and the UV90 wild-type to determine whether the UV90 mutation affects the expression of FRQ. Three circadian cycles were assayed every three hours, from 3 to 72 h in DD (constant darkness), and levels of FRQ protein were directly compared between the two strains. The pattern of FRQ expression in the wild-type (Figure 9A) was very similar to the pattern we previously reported for cultures on solid agar medium across two cycles from 24 to 69 h in DD [21]. The phosphorylation pattern of FRQ was similar in mutant and wild-type (Figure 9B). Expression of FRQ in the UV90 mutant was lower than wild-type in the first cycle and rapidly damped out such that expression was much lower in the second and third cycles (Figure 9A). Mean expression levels from three replicate experiments indicated that the level of FRQ expression in UV90 was significantly lower than in UV90^+^ (Figure 9C). We conclude that the UV90 mutation affects the functioning of the TTFL by damping the rhythm of FRQ protein. It is interesting to note that, although the levels of FRQ protein in the UV90 mutant were significantly lower than UV90 wild-type (Figure 9), there was very little effect on the period of the conidiation rhythm at the temperature used in this experiment (22°C, Figure 6; see also Table 1).

## Discussion

The UV90 mutation was found to affect two FRQ-less rhythms: the free-running rhythm under low-choline conditions (Figure 1A and 1C) and the heat-entrained rhythm in choline-sufficient conditions (Figure 7E–7H). Some *prd* mutations also affect more than one FRQ-less rhythm: The *prd-1* mutation affects FRQ-less rhythms in the *cel* [37] and *chol-1* [29] mutant backgrounds, in geraniol-supplemented cultures [17], and under heat entrained conditions [29], and *prd-2* affects the last three rhythms but has not been assayed for effects on *cel*. As with UV90, it is not known whether these *prd* mutations affect other FRQ-less rhythms that have not yet been assayed. Each of the products of the UV90, *prd-1* and *prd-2* genes may have multiple molecular targets and multiple functions, affecting several independent FLOs. Alternatively, if we assume the simplest model, that each gene product has one major function and one major target, then these results are consistent with the hypothesis that all FRQ-less rhythms are driven by a single FRQ-less oscillator, or FLO [29].

The various FRQ-less rhythms that have been reported appear in some cases to have different characteristics, and this may be seen to support the hypothesis of multiple independent FLOs. For example, temperature compensation is reported to be defective in some cases [14], [18], and in others the period is compensated within a temperature range but not outside of that range [16], [22]. This is similar to the observations of differences in temperature compensation between period-affecting *frq* mutants and other clock-affecting mutants in the presence of a functional FRQ/WCC TTFL, in which the genetic background can affect temperature compensation in *frq^+^* strains [38]. FRQ-less rhythms have been reported from several different laboratories, and the assay conditions, media, and genetic backgrounds of the strains differ. It is possible that under the widely varying conditions and genetic backgrounds used to assay the various FRQ-less rhythms, the properties observed may be affected by those conditions. The same suite of properties has not been assayed for all FRQ-less rhythms, and it is often impossible to directly compare them. For example, the molecular rhythm in DAG [25] has not been characterized beyond the observation that it persists in *frq-*less strains; the nitrate reductase rhythm [23] is known to persist in *frq* and *wc* null mutants and in both constant dark and constant light; but in neither case has temperature compensation been assayed nor is it known what molecular mechanisms drive these rhythms. In the absence of further details about the characteristics of the various FRQ-less rhythms, the most parsimonious assumption is that a single oscillator can drive all observed FRQ-less rhythms and this single FLO is affected by multiple gene products such as UV90, *prd-1* and *prd-2*. In the absence of a functional FRQ/WCC TTFL, the FLO may continue to oscillate with a low amplitude and with alterations of properties such as temperature compensation and period stability; the various conditions under which FRQ-less rhythms can be observed may act on different parameters of the FLO to increase its amplitude and allow FRQ-less rhythms with differing properties to be expressed.

Two lines of evidence suggest that the UV90 mutation primarily affects the FLO rather than the FRQ/WCC feedback loop. First, the free-running conidiation rhythm in low-choline *frq^10^* cultures is completely abolished by UV90 while a low-amplitude rhythm persists in the presence of functional *frq* (Figure 1). Second, entrainment to heat pulses is affected more strongly in the *frq^10^* background than in *frq^+^* (Figure 7). However, the UV90 mutation does have significant effects in the presence of functional *frq*. The amplitude of the free-running conidiation rhythm is dampened (Figure 1D), the temperature compensation of the free-running conidiation rhythm is affected (Figure 6), the amplitude of the oscillator as assayed by phase resetting is reduced (Figure 8) and the expression of FRQ protein is dampened (Figure 9). The UV90 gene product may have several molecular functions and may affect the FRQ/WCC TTFL independently of its effects on the FLO, but we prefer the simpler explanation stated previously that UV90 has a single major target. In this case, these findings lend support to the hypothesis that the FRQ/WCC feedback loop and the FLO interact with each other.

The long-period conidiation rhythm in the *chol-1* strain grown on choline-deficient medium persists in the absence of the FRQ/WCC TTFL, in either *frq* knockout or *wc* mutant strains [16]. This defines it as a FRQ-less rhythm driven by a FLO, but the long-period conidiation rhythm is also seen in *frq^+^* strains. This raises the question: What is happening to the FRQ/WCC TTFL when *frq^+^* cultures on low choline are producing long-period conidiation rhythms driven by the FLO? Shi *et al.* [28] found short-period rhythms of *frq* promoter activity in *chol-1* cultures with long-period conidiation rhythms, and concluded that the FLO in *chol-1* is “in no way connected to the circadian system” [28]. These authors did not use mathematical techniques to look for relative coordination or frequency demultiplication between the short-period molecular rhythm and the long-period conidiation rhythm and so might have missed evidence of interactions. We have previously reported evidence for an influence of the FRQ/WCC TTFL on the FLO in *chol-1*: a short-period *frq^1^* mutation [39] and a *wc-2* mutation [16] can significantly alter the long period in *chol-1*, and introducing the *frq^10^* mutation into *chol-1* can lengthen the long period (Table 1 and [29]).

If our suggestion that the UV90 mutation primarily affects the FLO is correct, then our results now provide evidence for an influence of the FLO on the FRQ/WCC TTFL. The UV90 gene product is required for normal functioning of the FLO and to sustain the amplitude of the FRQ/WCC TTFL. Evidence that *prd* mutations can affect rhythmicity in both *frq^+^* and *frq^10^* backgrounds [29] suggests interactions between these two oscillators. The complete system maybe more complex, with multiple targets for the UV90 and *prd* gene products, and multiple oscillators, but the current data set can be explained with this two-oscillator model. Further experiments will be needed clarify the functions of the gene products in question, identify their targets, and describe the mechanism of the FLO(s).

We conclude that the simplest model for the architecture of the circadian system of *Neurospora* [29] is a single FLO that mutually interacts with, and is required to support, the FRQ/WCC TTFL. The fungal circadian system may thus be conceptually similar to the cyanobacterial system, in which a post-translational core oscillator interacts with a TTFL [6]. The coupling of a TTFL with a post-transcriptional oscillator is an emerging theme in circadian biology [40], with examples coming from plants [41], [42] and animals [8], [43] as well as the cyanobacteria. Mathematical modeling demonstrates that such a system of coupled oscillators could be more robust to noise and changes in growth rate than either oscillator alone [6], [44]. *Neurospora* has long been a fruitful model system for elucidating mechanisms of circadian rhythmicity common to many organisms, and therefore our results should encourage the search for circadian system components outside of the TTFL loops in other eukaryotic organisms.

## Materials and Methods

### Strains and growth conditions

All strains carried the *ras-1^bd^* (formerly *bd*) and *csp-1* mutations, as previously described [15], [29]. The *frq^10^* mutation is a knockout created by targeted gene disruption [12]. The *chol-1* mutation requires choline for normal growth as previously described [16], [45]. Multiple mutant strains were created by standard crossing methods, and were backcrossed to the *ras^bd^* strain to verify genotypes by segregation of the expected phenotypes. Cultures were grown on maltose/arginine (MA) medium containing Vogel's salts, 0.5% maltose, 0.01% arginine, 2% agar, and either high choline (100 µM) or no choline supplementation. On 100 µM choline, the *chol-1* strains are not distinguishable from *chol^+^* strains [39]. Cultures were initially grown in constant light on agar plates, and small plugs of mycelium were transferred to race tubes (for rhythm assays) or to cellophane-covered Petri plates (for biochemical assays) as previously described [21], [29] before transfer to constant darkness (DD) at 22°C.

### Mutagenesis

The parent strain for mutagenesis was genotype *csp-1*; *chol-1 ras^bd^*; *frq^10^*. Uninucleate microconidia were prepared by the method of Maheshwari [46], [47], using iodoacetate to induce microconidiation. Microconidia were suspended in water and exposed to ultraviolet light. Survival rates (compared to untreated microconidia) ranged between 10% to 80% in various experiments. Treated microconidia were plated on a sorbose-containing medium to induce colonial growth and individual colonies were tested for rhythmic phenotypes on race tubes.

### Temperature compensation

All strains carried the wild-type *frq^+^* allele (in addition to *ras-1^bd^* and *csp-1*) and either *chol^+^* or *chol-1*, and UV90^+^ or UV90. The four genotypes used were: (1) *csp-1*; *ras^bd^*, (2) *csp-1*; *ras^bd^*; UV90, (3) *csp-1*; *chol-1 ras^bd^*, and (4) *csp-1*; *chol-1 ras^bd^*; UV90. Cultures were grown on race tubes containing MA medium with 100 µM choline. The period of the conidiation rhythm and the growth rate were assayed for each tube by linear regression as previously described [29], [39]. At 19°C the UV90 strains produced only two or three conidiation bands before the rhythm damped out, therefore only the second period on each tube was used for all strains at this temperature. The number of tubes averaged for means ranged between 6 and 12. Means were compared using the two-tailed Student's t-test for samples with equal variances. UV90 strains were compared to the corresponding UV90^+^ strains, i.e., *chol^+^* UV90 was compared to *chol^+^* UV90^+^, and *chol-1* UV90 was compared to *chol-1* UV90^+^.

### Entrainment to high temperature pulses

All strains carried *chol-1* (in addition to *ras-1^bd^* and *csp-1*) and either *frq^+^* or *frq^10^*, and UV90^+^ or UV90. The four genotypes used were: (1) *csp-1*; *chol-1 ras^bd^*, (2) *csp-1*; *chol-1 ras^bd^*; UV90, (3) *csp-1*; *chol-1 ras^bd^*; *frq^10^*, and (4) *csp-1*; *chol-1 ras^bd^*; *frq^10^*; UV90. Cultures were grown in DD at 22°C on race tubes containing MA medium with 100 µM choline to repair the *chol-1* defect. 2-hour heat pulses to 32°C were delivered as previously described [15], [29] and density traces were collected and analysed as previously described [15], [29]. Average density traces were calculated from the last two complete cycles in each experiment, averaging two cycles per tube and six tubes per set for N = 12. Standard errors were calculated for each average pixel value. Average density profiles were normalized by setting the minimum and maximum pixel values to 0 and 1, and confidence intervals were plotted as ± one S.E.M.

### Phase resetting assays

All strains carried the wild-type alleles of *chol^+^* and *frq^+^* (in addition to *ras-1^bd^* and *csp-1*). The two genotypes used were: (1) *csp-1*; *ras^bd^*, and (2) *csp-1*; *ras^bd^*; UV90. Cultures were grown in DD at 22°C on race tubes containing MA medium. For light resetting, groups of 4 tubes were exposed to a 2 min pulse of cool-white fluorescent light at 20–24 µmol/m^2^/s at 3 h intervals between 24 to 39 h in DD. For heat pulse resetting, groups were exposed to a 15 min pulse of 37°C at 3 h intervals between 22 to 40 h in DD. After growth had finished at 22°C, the positions of the bands that formed after the pulses were used to calculate the phase (in circadian time) of each race tube relative to the average phase of the un-pulsed control group. Circadian time (CT) was calculated using the periods determined from the un-pulsed controls, defining one period (approx. 22 h) as equal to 24 circadian hours. One circadian hour is therefore 1/24^th^ of a period. CT 12 is defined as the light-to-dark transition. Phase shifts were calculated as the difference in circadian hours between the pulsed tubes and the average phase of the un-pulsed controls, and were plotted against the CT of the pulses to generate phase response curves. To generate phase transition curves, the new phases of the pulsed tubes (in CT) were plotted against the CT of the pulses, defined as the phases (in CT) of the controls (“old phase”) at the time of the pulses.

### Western blotting for FRQ expression

The method was essentially as previously described [21]. Cultures of the *ras^bd^*; *csp-1*; *chol-1* and *ras^bd^*; *csp-1*; *chol-1* UV90 strains were grown at 22°C on top of cellophane on MA medium with 100 µM choline in 150 mm Petri plates. Cultures were initially grown in constant light, then transferred to DD, and harvested after 72 hours of total growth. Circadian phase (reported as hours in darkness) was varied by varying the time at which cultures were transferred from light to dark. Plates were transferred every three hours, and samples were collected from 3 to 72 h in DD. Cultures were harvested, protein was extracted, and FRQ was detected as previously described [21]. The primary antibodies were generously supplied by M. Merrow [48] and M. Brunner. Samples were divided into three sets for electrophoresis and blotting: 3–27, 27–48, and 48–72 h in DD. Both UV90^+^ and UV90 samples were run on the same gel to directly compare expression levels between strains. FRQ was normalized against total protein by staining blots with Coomassie Blue after immunodetection. To plot one complete time series from 3 to 72 h, normalized values were adjusted to equalize the repeated samples between sets (27 and 48 h). To calculate mean ratios, three time points were chosen near the peak values in each cycle. Three independent experiments were carried out at each time point, running UV90^+^ and UV90 samples on the same gel for direct comparison of FRQ levels. The relative level of FRQ in UV90 was divided by the relative level in UV90^+^ to calculate the ratio. A ratio of 1.00 indicates that FRQ levels are the same in the UV90^+^ and UV90 samples. The means and S.E.M.s of the three independent ratios were calculated. One-tailed one sample t-tests were used to test the null hypothesis that the means are not less than 1.00, that is, that UV90 FRQ levels are not lower than UV90^+^ levels.
